# The use of the paramedian forehead flap alone or in combination with other techniques in the reconstruction of periocular defects and orbital exenterations

**DOI:** 10.1038/s41433-022-01985-9

**Published:** 2022-03-03

**Authors:** Terence W. Ang, Valerie Juniat, Micheal O’Rourke, James Slattery, Brett O’Donnell, Alan A. McNab, Thomas G. Hardy, Yugesh Caplash, Dinesh Selva

**Affiliations:** 1 Discipline of Ophthalmology and Visual Sciences, The University of Adelaide, Adelaide, SA Australia; 2 South Australian Institute of Ophthalmology, Royal Adelaide Hospital, Adelaide, SA Australia; 3 Department of Ophthalmology, Royal Adelaide Hospital, Adelaide, SA Australia; 4 Orbital, Plastic and Lacrimal Clinic, Royal Victorian Eye and Ear Hospital, East Melbourne, Vic Australia; 5 Department of Ophthalmology, Royal North Shore Hospital, Sydney, NSW Australia; 6 Centre for Eye Research Australia, University of Melbourne, Melbourne, Vic Australia; 7 Department of Surgery, Royal Melbourne Hospital, University of Melbourne, Melbourne, Vic Australia; 8 Department of Plastic and Reconstructive Surgery, Royal Adelaide Hospital, Adelaide, SA Australia

**Keywords:** Health care, Education

## Abstract

**Purpose:**

The paramedian forehead flap (PMFF) is a reconstructive option for large eyelid defects and orbital exenterations. We report a series of cases where PMFF reconstruction was carried out at various institutions in Australia.

**Methods:**

This study was a multi-centre, retrospective, non-comparative case series investigating the clinical outcomes of the PMFF for reconstructing periocular defects and orbital exenterations.

**Results:**

This case series describes twenty-seven patients (Female = 15, Male = 12), operated between 1991 to 2019, with a median age of 81 years (range: 45–93 years). Defect locations involved combinations of the medial canthus (16/27, 59.3%), upper eyelids (7/27, 25.9%), lower eyelid (4/27, 14.8%), both upper and lower eyelids (5/27, 18.5%), and orbital (7/27, 25.9%). There were no cases of flap necrosis. Minor post-operative complications were observed in ten patients with the most common being lagophthalmos. Median duration of follow-up was 17months (Range: 2months- 23years).

**Conclusions:**

The PMFF is a versatile reconstructive tool for a range of periocular defects and orbital exenterations with minor post-operative complications.

## Introduction

Reconstruction of large eyelid and orbital defects can be challenging in head and neck surgery, due to the orbit’s three-dimensional complexity. Reconstruction of periorbital defects aims to provide ocular protection, eyelid function, and acceptable cosmesis [1]. Reconstructive options may include harvesting flaps from the scalp, forehead, galea, and pericranium. We describe the paramedian forehead flap (PMFF) technique and report our results of using the PMFF in reconstructing large eyelid defects and orbital exenterations.

## Surgical anatomy and technique

The PMFF is a rotational locoregional skin flap harvested from the forehead. The forehead is supplied by the supraorbital plexus, consisting of the dorsal nasal, supratrochlear and supraorbital arteries. The PMFF is primarily supplied by the supratrochlear artery, a terminal branch of the ophthalmic artery. The supratrochlear artery courses superficially to the frontalis muscle, approximately 2 cm lateral to midline, and terminates in the subdermal plexus. The dorsal nasal and supraorbital vessels provide secondary blood supply to the forehead [2, 3].

**Fig. 1:**
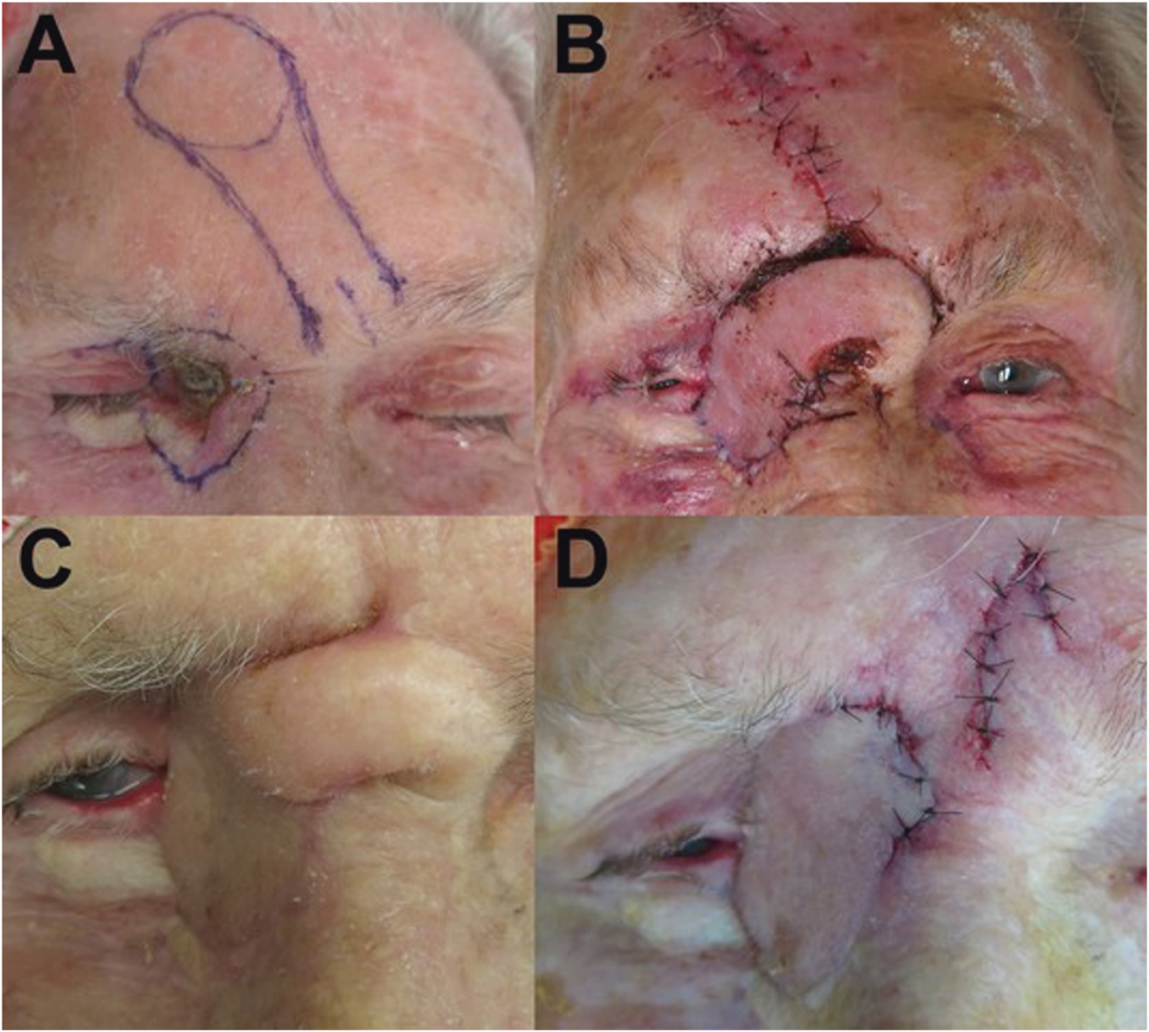
Two-stage contralateral PMFF repair following excision of a right recurrent medial canthus BCC (Case 24). **A** Right medial canthus BCC and the pre-operative outline of the flap for the first stage of repair. **B** Closure of the medial canthus defect with the pedicled flap and direct closure of the forehead donor site. **C** shows the flap pedicle crossing the glabella prior to division in the second stage. **D** Pedicle division and flap inset at the right medial canthus occurred five weeks after the first stage. Legend: PMFF- Paramedian Forehead Flap; BCC- Basal cell carcinoma.

The PMFF can be completed within three stages. Firstly, the identified supratrochlear vessels are mapped via palpation or Doppler ultrasound. Up to 8% of intact supratrochlear arteries cannot be identified by palpation or Doppler imaging, in which case alternative reconstructive options should be considered [4]. The PMFF dimensions require consideration prior to harvest and insertion. Harvested flaps can be as wide as 6 cm, but the hairline limits flap length [1]. The pedicle base thickness determines many of the flap characteristics; with a thinner pedicle potentially compromising viability, and a thicker pedicle restricting flap rotation and causing kinking [4]. The flap should be thinned to match the defect depth (e.g., thinning to the subdermal layer is appropriate for eyelid defects, whilst orbital exenteration defects may not require thinning) [1]. A foil template is based on the corresponding intact contralateral tissue. The template is marked to the defect’s exact dimensions to prevent loss of surface detail and contractures with the collapse of underlying structures (Fig. 1A) [4].

After flap incision, blunt dissection begins distally, leaving periosteum over the frontal bone. The flap is then elevated off the subgaleal (subfascial) plane and rotated to the defect site [4, 5]. Excessive tension at closure can cause poor perfusion, leading to flap necrosis. If blanching or venous congestion is noted at the time of closure, releasing sutures reduces tension and permits secondary healing [4]. Tension-free closure, particularly for medial canthus defects, also prevents distortion of the eyelids, to achieve good aesthetic and normal tear flow [6]. Donor site closure should occur near to the midline and can be achieved with a combination of direct closure; split-thickness skin graft, or secondary intention (Fig. 1B) [1, 4, 5, 7].

After 2–3 weeks, the pedicle crossing the skin of the nose requires division (Fig. 1C) [8]. However, successful pedicle division occurring as early as 1 week has been reported [9]. Indocyanine green angiography can help assess distal perfusion prior to division [10]. Firstly, the distal and proximal portions of the flap are marked. The proximal portion is marked with an inverted “V”, allowing linear closure of the brow (Fig. 1D). Debulking the cephalic aspect of the distal flap can help create appropriate contouring, but the distal flap should remain inset to prevent vascular compromise [4].

The PMFF may utilise the contralateral or ipsilateral supratrochlear vessels. Additionally, single-stage techniques may involve tunnelling a de-epithelised flap under the skin at the radix, negating future flap division [5, 11, 12]. In specific patient populations, particularly smokers, excessive thinning of the PMFF should be avoided due to poor vascularity, and surgical delay improves flap survival [5, 13]. A delay between fashioning the PMFF and flap inset may also be indicated in situations including a previous significant scar within the proposed flap territory, uncertain vascular supply from previous injury, complex extensions of the flap, the flap design extending across multiple vascular territories, or a history of facial radiation. When delaying the PMFF, the template is first incised to the periosteum without elevation. A thin peripheral outline is left intact, ensuring flap survival, and transfer and inset occurs approximately 3–4 weeks later [5].

## Method

This study was a multi-centre, retrospective, non-comparative case series investigating the clinical outcomes of the PMFF for the reconstruction of periocular defects and orbital exenterations. Inclusion criteria consisted of any periocular defect or orbital exenteration reconstructed using the PMFF, either in combination with other forms of reconstruction, or as the sole technique.

Patients were identified from the Oculoplastics and Plastic Surgery Unit at the Royal Adelaide Hospital (Adelaide, Australia), Royal Victorian Eye and Ear Hospital (Melbourne, Australia), Royal Melbourne Hospital (Melbourne, Australia), and Royal North Shore Hospital (Sydney, Australia). Data was sourced from electronic medical records and paper notes. Data collected included: patient demographics (gender, age, smoking status, comorbidities, anticoagulants/antiplatelets usage and prior periocular procedures), diagnosis, surgical details (defect location, depth, and dimensions, number of reconstructive stages, flap rotation, flap thickness, pedicle width, plane of dissection, concurrent procedures of defect reconstruction, donor site closure, intraoperative complications and length of admission), and post-operative details (complications, follow-up). All research was conducted in accordance with the Declaration of Helsinki. The research protocol was approved by the Royal Adelaide Hospital Human Research Ethics Committee with a waiver of consent granted.

The literature review was conducted using PubMed, Google Scholar, and Embase. Keywords included: orbital exenterations, eyelid defects, peri-orbital/orbital reconstruction, forehead flaps, and paramedian forehead flaps. Reference lists were reviewed, and further relevant papers were included.

## Results

**Fig. 2:**
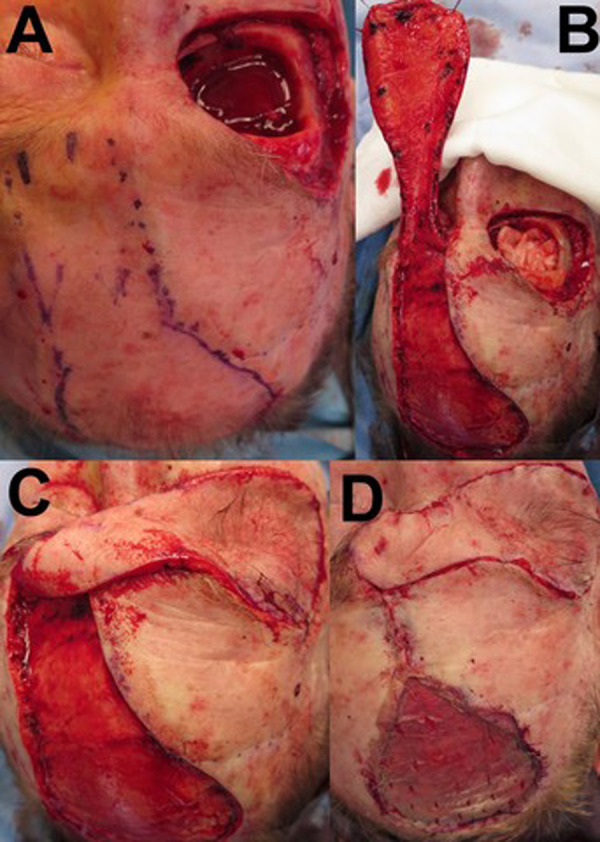
A single-stage contralateral PMFF following an orbital exenteration for a primary SCC (Case 1). **A** demonstrates the exenterated orbit and the PMFF outlined prior to incision. **B** shows the PMFF elevated off the pericranium. **C** the PMFF is rotated into the orbital defect. **D** demonstrates the flap inset at the orbit and the use of a STSG for the forehead donor site. Legend: PMFF- Paramedian Forehead Flap; SCC- Squamous cell carcinoma; STSG- split-thickness skin graft.

This case series describes twenty-seven patients (Female = 15, Male=12), operated between 1991 and 2019. The median age at time of procedure was 81 years (range: 45–93 years). 5/27 (18.5%) patients were smokers, and 5/27 (18.5%) patients continued their preoperative antiplatelets/anticoagulants. Relevant co-morbidities include cardiovascular/vascular disease (12/27, 44.4%), Type 2 diabetes mellitus (3/27, 11.1%) and previous periocular radiotherapy (2/27, 7.4%). Thirteen patients had prior periocular procedures including: previous tumour excisions (10/27, 37.0%), flap/graft reconstructions (6/27, 22.2%), ectropion repair (1/27, 3.7%), orbital exenteration (1/27, 3.7%) (Fig. 2) and ptosis correction (1/27, 3.7%).

One patient had reconstruction following a severe facial abscess leading to tissue loss related to previous complete SCC excision (1/27, 3.7%). The remainder of patients had reconstruction following excision of tumours including basal cell carcinomas (17/27, 63.0%), squamous cell carcinomas (7/27, 25.9%), metastatic anaplastic meningioma (1/27, 3.7%), and an anaplastic carcinoma of lacrimal gland (1/27, 3.7%). 16/27 (59.2%) patients had primary tumours, whilst 10/27 (37.0%) patients had recurrent tumours.

Patients had defects affecting single or multiple locations. Defect locations involved combinations of the medial canthus (16/27, 59.3%), upper eyelids (7/27, 25.9%), lower eyelid (4/27, 14.8%), both upper and lower eyelids (5/27, 18.5%), and orbital (7/27, 25.9%). Defect depth was to the bone in all eighteen patients recorded. Defect dimensions were recorded for sixteen patients and ranged from 12x12mm to 30x50mm. Three patients had orbital exenterations (3/27, 11.1%).

All patients (27/27, 100%) had a general anaesthetic and preoperative identification of the supratrochlear vessels was achieved via doppler ultrasound. Reconstruction was completed in one stage (6/27, 22.2%), two stages (17/27, 63.0%) or three stages (4/27, 14.8%). One patient was initially planned for a two-stage procedure, but only underwent the first stage as their co-morbidities made them unsuitable for further procedures. Flap rotation involved contralateral (17/27, 63.0%), ipsilateral (7/27, 25.9%) and central/median (3/27, 11.1%) approaches. Average pedicle width, reported for five patients, was 12 mm (range: 10–15 mm), but pedicle length was not reported. The dissection plane for six patients included subperiosteal (4/6, 66.7%) and subgaleal (2/6, 33.3%). Dissection plane was not recorded for the remaining patients. Flap inset at the defect site occurred with a combination of nylon, vicryl/monocryl and/or gut sutures, depending on the depth and contours of the defect.

Fifteen patients also had concurrent reconstructive procedures ranging solely or in combination of other reconstructive flaps (9/27, 33.3%), grafts (6/27, 22.2%), tarsorrhaphy (1/27, 3.7%) and upper cheek lift (1/27, 3.7%). Donor site closure was achieved solely by or a combination of direct closure with 3-0, 4-0 or 5-0 vicryl/moncryl and/or nylon (17/27, 63.0%), skin grafts (7/27, 25.9%) and secondary intention (7/27, 25.9%). Skin grafts were harvested from either the supraclavicular area or lateral thigh. Six patients underwent a day procedure (6/27, 22.2%), with the remaining admitted (21/27, 77.8%) for a median duration of 6 days (range: 1–22 days).

One patient on warfarin experienced intraoperative bleeding and developed a post-operative haematoma, which was managed conservatively. Post-operative complications were observed in ten patients and ranged from lagophthalmos (6/27, 22.2%) which was treated either conservatively (e.g., topical lubricants) or with subsequent surgery (e.g., lid recession surgery); eyelid malposition (2/27, 7.4%) treated surgically, ptosis (2/27, 7.4%) managed conservatively; pre-septal cellulitis (1/27, 3.7%) treated with antibiotics; a radionecrotic ulcer (1/27, 3.7%) treated with antibiotics; flap trapdoor/pin-cushioning of medial upper lid (1/27, 3.7%) managed with massage; ectropion (1/27, 3.7%) managed with a skin graft; and a naso-cutaneous fistula (1/27, 3.7%) treated with incision and closure. Median duration of follow-up was 17 months (Range: 2 months- 23 years). Selected cases with reported complications are summarised in Table 1.

**Table 1:** Summary of selected PMFF cases with complications.

Case	Gender/Age	Defect location	Reconstructive approach					Complications & Management	Follow-up (months)
			Stages	Laterality	Concurrent procedure	Donor site closure	Subsequent stages (after first stage)		
**2**	F/88	Medial canthus	2	Contralateral	Upper cheek lift	FTSG	Flap division, with thinning and inset into position at 4 weeks	Pre-septal cellulitis, radionecrotic ulcer, both managed with antibiotics	30
**5**	F/86	Medial canthus	2	Ipsilateral	None	Direct closure	Flap division and inset at 3 weeks	Eyelid malposition, naso-cutaneous fistula, both managed surgically	4
**6**	F/50	Medial canthus	2	Contralateral	None	Secondary intention	Flap division and further tumour excision at 2 weeks	Lagophthalmos managed conservatively	5
**9**	M/91	Upper and lower eyelids; medial canthus	2	Contralateral	Mucosal graft to under surface of PMFF	FTSG	Flap division at 5 months	Lagophthalmos managed conservatively	10
**14**	M/67	Upper eyelid; orbit	2	Contralateral	None	FTSG	FTSG to upper lid at 14 months for lagophthalmos	Lagophthalmos, ptosis, both managed conservatively	144
**16**	M/83	Upper eyelid; orbit; medial canthus	2	Ipsilateral	FTSG	Direct closure	Pedicle revision at 3 months	Ptosis managed conservatively	6
**18**	F/91	Orbit	1	Ipsilateral	None	Direct closure	None	Lagophthalmos managed with lid recession surgery	64
**19**	F/93	Medial canthus; both upper and lower medial eyelids	1	Ipsilateral	Posterior lamella reconstruction with FTSG and periosteal flap	Direct closure	None	Pin cushioning of medial upper lid, lagophthalmos, both managed conservatively	9
**20**	F/82	Medial canthus; lower eyelid	2	Contralateral	Tenzel flap for lower lid	Direct closure + secondary intention	Flap division at 4 weeks	Ectropion managed with FTSG	68
**24**	F/90	Medial canthus	2	Contralateral	None	Direct closure	Flap division at 5 weeks	Lagophthalmos managed with medial canthoplasty	60

*F*- Female; *FTSG*- Full-thickness skin graft; *M*- Male; *PMFF*- Paramedian Forehead Flap; *STSG*-Split-thickness skin graft.

## Discussion

The PMFF is an effective option for periorbital defects too small for free flaps and too large for repair with an eyelid flap [1]. PMFF usage in oculoplastics has been mostly limited to large eyelid defects, primarily affecting the lower eyelid and medial canthus [8, 14]. The PMFF is also a suitable option for reconstructing defects following exenteration, closure of sinonasal fistulas, or other communications between the orbit and adjacent cranial or sinus spaces (Fig. 2, 3A) [1, 5, 15–18].

**Fig. 3:**
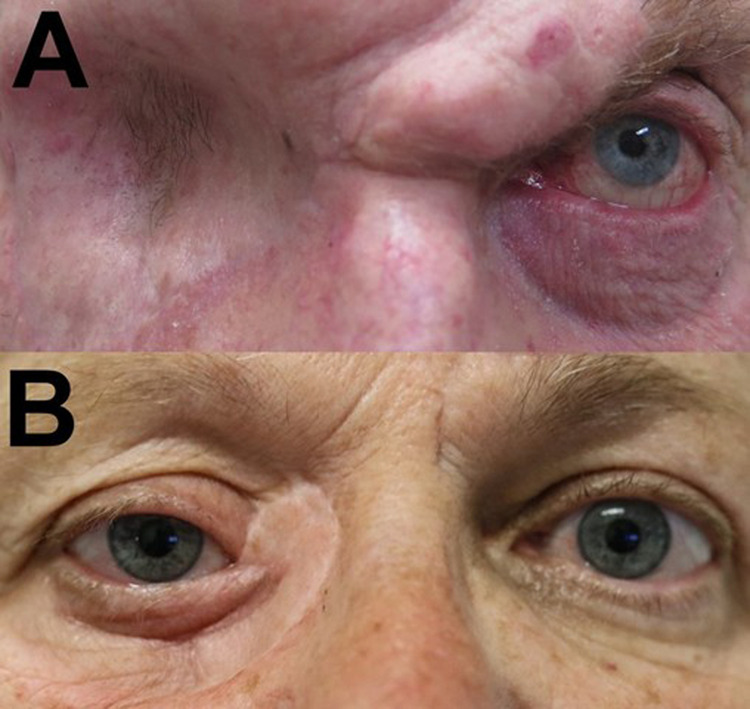
Post-operative outcomes of a PMFF for peri-ocular defects and exenterations. **A** 3-months after a single-stage PMFF following an orbital exenteration for a primary SCC (Case 1). **B** 24-months after a two-stage contralateral PMFF following excision of a primary SCC at the right medial canthus (Case 4). Legend: PMFF- Paramedian Forehead Flap; SCC- Squamous cell carcinoma.

The PMFF is a viable option for patients with a range of different comorbidities, particularly those that would normally predispose to poor wound healing, such as pre-operative radiotherapy [5]. In our series, patients who had various vascular risk factors (e.g., active smoker, diabetes, etc) had a successful PMFF with minimal complications and no flap failure.

PMFF reconstruction allows adequate restoration or maintenance of vision, eyelid function, and cosmesis. The proximity of the flap matches skin colour, tissue texture and structure between the local donor and recipient site [1, 18, 19]. Additionally, the pliable PMFF can be thinned and trimmed to conform to the defect’s complex contours (Fig. 3B). The successful repair of the medial orbital wall via de-epithelisation of the distal half of the flap was demonstrated in our cases. A single flap can be constructed to cover multiple periorbital defects/subunits (e.g., medial canthus, medial eyebrow, and nasal radix) [6, 20]. Additionally, splitting the PMFF can help simultaneously recreate upper and lower eyelids and this was demonstrated successfully within our cases [21].

Conventional methods utilise a contralateral PMFF, achieving a large rotation arc, increasing flap extension, and reducing risk of vascular compromise. However, bulging of the nose dorsum occurs as the pedicle crosses the upper nose [7]. An ipsilateral PMFF can reduce glabella bulkiness and distal flap necrosis, which are important factors influencing flap survival rates. In Kim et al.’s case series, all ipsilateral PMFFs for medial canthus reconstruction survived without any significant flap bulk. The pedicle arc of the ipsilateral PMFF was rounded to prevent potential venous congestion, which can occur with more acute approaches [7].

Within our case series, a contralateral rotation was more common and there were no cases of venous congestion reported for patients who underwent an ipsilateral PMFF. There were no discrepancies of the complication rate between the rotations. A single-stage, tunnelled median forehead flap has been described for repair of medial canthal and eyelid defects. This technique was similarly able to minimise glabellar bulkiness, whilst maintaining the natural surface contour of the medial canthus [22]. Within our case series, a single-stage PMFF was also achieved following an orbital exenteration via turning the flap into the defect and small width of the right medial orbital skin excised to allowed bridging of the pedicle. Nevertheless, despite use of both contralateral, ipsilateral and median rotations, glabellar bulkiness was not a reported issue within our case series.

The PMFF is a reliable method with minimal donor site morbidity, a dependable blood supply, which does not require any microvascular surgical techniques [1, 18]. Common complications reported include distal flap necrosis, infection, poor aesthetic outcome, and sensory loss at the forehead [4, 7, 8, 16, 23]. Sensory loss at the forehead has been reported to be temporary due to the overlapping adjacent supraorbital nerve territory [16]. Lower eyelid retraction with inferior scleral show has also been reported [24]. Lagophthalmos was the most common complication noted within our case series, which is not widely reported in the literature. Within our case series, there were no flap failures or distal necrosis, and post-operative complications were minor.

The high vascularity of the PMFF facilitates healing and early adjuvant radiotherapy, which is well tolerated [1, 16]. This was consistent with our findings, however, we noted the occurrence of a radionecrotic ulcer over the flap in one patient that healed with appropriate management. Furthermore, the rich distal blood supply of the flap facilitates take of mucosal or skin grafts to help restore the conjunctiva in full-thickness eyelid defects [23].

Our case series also confirms that successful flap division can occur as early as 1-2 weeks despite underlying comorbidities (e.g., Type 2 diabetes mellitus). Nevertheless, a PMFF may be planned as a multi-staged approach, requiring patients to be medically fit to undergo multiple surgeries [4]. Co-morbidities or complications during admission may limit the patient from proceeding to the next stage of reconstruction.

Details of post-operative outcomes and duration of follow-up varied in the literature and case series. There was also a lack of measurable criteria or external assessment by independent peers to review patient outcomes (e.g., cosmesis and functional assessment). Applying standardised objective measures can be inherently difficult as cases are often complex. Within our case series, specific flap characteristics were not widely recorded (e.g., flap dimensions, thickness, and pedicle width).

In conclusion, the PMFF is a versatile reconstructive tool for a range of periocular defects and orbital exenterations. The literature and our case series have demonstrated that the PMFF has favourable characteristics, notably high vascularity, pliability, and potential for surgical adaptations, facilitating successful restoration of periocular/orbital form and function, with minor complications.

### Summary

#### What was known before

- The PMFF has historically been used for repairing a variety of facial defects, particularly nasal defects.
- The PMFF can also be applied to periocular reconstruction and orbital exenterations. However, such cases are usually represented as part of broad case series within head and neck surgery.

#### What this study adds

- Large case-series of PMFF specifically within the reconstruction of periocular defects and orbital exenterations. This outlines the favourable characteristics of this flap and complications that may be encountered.
- A review of the literature regarding these applications in oculoplastic reconstruction.
